# Identifying the Active Ingredients of a Computerized Speech and Language Therapy Intervention for Poststroke Aphasia: Multiple Methods Investigation Alongside a Randomized Controlled Trial

**DOI:** 10.2196/47542

**Published:** 2023-12-05

**Authors:** Madeleine Harrison, Rebecca Palmer, Cindy Cooper

**Affiliations:** 1 Sheffield Centre for Health and Related Research University of Sheffield Sheffield United Kingdom

**Keywords:** aphasia, stroke, computer therapy, tele-rehabilitation, speech and language therapy, word finding, qualitative, language, language therapy, speech therapy, aphasia therapy, speech, interview, self managed, computer aphasia, persistent aphasia, rehabilitation, machines, technology, computer, online, online health, ehealth, digital health

## Abstract

**Background:**

Aphasia is a communication disorder affecting more than one-third of stroke survivors. Computerized Speech and Language Therapy (CSLT) is a complex intervention requiring computer software, speech and language therapists, volunteers, or therapy assistants, as well as self-managed practice from the person with aphasia. CSLT was found to improve word finding, a common symptom of aphasia, in a multicenter randomized controlled trial (Clinical and Cost Effectiveness of Computer Treatment for Aphasia Post Stroke [Big CACTUS]).

**Objective:**

This study provides a detailed description of the CSLT intervention delivered in the Big CACTUS trial and identified the active ingredients of the intervention directly associated with improved word finding for people with aphasia.

**Methods:**

We conducted a multiple methods study within the context of a randomized controlled trial. In study 1, qualitative interviews explored key informants’ understanding of the CSLT intervention, how the components interacted, and how they could be measured. Qualitative data were transcribed verbatim and analyzed thematically. Qualitative findings informed the process measures collected as part of a process evaluation of the CSLT intervention delivered in the Big CACTUS trial. In study 2, quantitative analyses explored the relationship between intervention process measures (length of computer therapy access; therapists’ knowledge of CSLT; degree of rationale for CSLT tailoring; and time spent using the software to practice cued confrontation naming, noncued naming, and using words in functional sentences) and change in word-finding ability over a 6-month intervention period.

**Results:**

Qualitative interviews were conducted with 7 CSLT approach experts. Thematic analysis identified four overarching components of the CSLT approach: (1) the StepByStep software (version 5; Steps Consulting Ltd), (2) therapy setup: tailoring and personalizing, (3) regular independent practice, and (4) support and monitoring. Quantitative analyses included process and outcome data from 83 participants randomized to the intervention arm of the Big CACTUS trial. The process measures found to be directly associated with improved word-finding ability were therapists providing a thorough rationale for tailoring the computerized therapy exercises and the amount of time the person with aphasia spent using the computer software to practice using words in functional sentences.

**Conclusions:**

The qualitative exploration of the CSLT approach provided a detailed description of the components, theories, and mechanisms underpinning the intervention and facilitated the identification of process measures to be collected in the Big CACTUS trial. Quantitative analysis furthered our understanding of which components of the intervention are associated with clinical improvement. To optimize the benefits of using the CSLT approach for word finding, therapists are advised to pay particular attention to the active ingredients of the intervention: tailoring the therapy exercises based on the individual’s specific language difficulties and encouraging people with aphasia to practice the exercises focused on saying words in functional sentences.

**Trial Registration:**

ISRCTN Registry ISRCTN68798818; https://www.isrctn.com/ISRCTN68798818

## Introduction

Globally, an estimated 12.2 million cases of stroke occur each year [1]. Approximately one-third of stroke survivors experience aphasia, an acquired communication disorder, affecting the production and comprehension of verbal language and the ability to read or write [2]. Due to health care costs, limited speech and language therapy is provided to people with poststroke aphasia beyond the first few months [3].

Self-managed approaches have gained traction as health care providers try to meet the growing demand for their services [4]. Computerized Speech and Language Therapy (CSLT) provides an opportunity for people with aphasia to self-manage their own rehabilitation by practicing rehabilitation exercises in their own homes [5]. A review demonstrated that CSLT is effective compared to no therapy but acknowledged the need for more research to establish whether it is effective compared to face-to-face speech and language therapy [6].

The Clinical and Cost Effectiveness of Computer Treatment for Aphasia Post Stroke (Big CACTUS) trial evaluated the clinical effectiveness and cost-effectiveness of CSLT for people with poststroke aphasia in the long term compared to usual care or attention control. Full details of the trial have been reported elsewhere [5,7]. CSLT was found to improve word finding compared to usual care and attention control, although the clinical gains did not generalize to improvement in conversation. CSLT was found to enable greater amounts of practice than available with usual care (28 hours CSLT vs 3.8 hours usual speech and language therapy between baseline and 6 months), leading to improved ability to retrieve words of personal importance than with usual care (CSLT group improved word finding by 16.2% [*P*<.0001] more than those in the usual care group and 14.4% [*P*<.0001] more than those in the attention control group). Demonstrating that CSLT can be used to support sufficient amounts of therapy practice to enable effective retrieval of specific words, with speech and language therapists (SLTs) needing to provide additional support and activities to help people with aphasia use the words in everyday contexts [5,7].

CSLT is a complex rehabilitation intervention often requiring technology or software relevant to the impairment of people with aphasia, input and support from professionals or informal caregivers, as well as a commitment from people with aphasia to use the CSLT. The complexity of CSLT arises from its multiple components and actors that operate both independently and interdependently. This can make it difficult to identify the components or groups of interrelated components that are important mechanisms of change [8]. Intervention components may include the content of an intervention, features that promote adherence, or aspects of implementation [9]. The importance of identifying the active ingredients (the essential and indispensable aspects) of interventions has been recognized in aphasia research [10] and beyond [11].

Furthermore, complex interventions are typically implemented within complex systems by individuals with their own competing views and objectives, which can often result in intervention adaptation at the point of implementation [12]. If we do not know which components of the CSLT approach are particularly associated with improved word retrieval and SLTs only implement certain components, but not those most closely associated with improvement, this may limit the potential benefits to people with aphasia [13]. By establishing which CSLT approach components are associated with improved word retrieval, we can provide additional information to SLTs considering using the approach in their practice to help them optimize the way in which they implement it [13].

A comprehensive understanding of complex interventions is also vital to enable better reporting of interventions. The introduction of the Template for Intervention Description and Replication (TIDieR) checklist and guide [14] has both encouraged researchers to provide more detail about their interventions but also acted as an evaluation tool to demonstrate how infrequently intervention details are reported. Intervention reporting has been found wanting in both aphasia [15] and telehealth research [16].

In this study, we aimed to provide a detailed description of the CSLT approach components delivered in the Big CACTUS study and identify those components most associated with change in word-finding ability for people with aphasia, by addressing the following 2 questions:

1. What are the components of the CSLT intervention delivered in the Big CACTUS trial?
2. Are any of the components directly associated with improvement in word finding?

## Methods

### Overview

From October 2014 to August 2016, people with aphasia were recruited into a pragmatic, 3-arm, single-blind (outcome assessor), individually randomized controlled trial of CSLT [5,7]. The Big CACTUS trial was carried out in 21 SLT departments across the United Kingdom. Sites were recruited through national advertisements via relevant professional bodies. Participants were eligible if they had received a diagnosis of poststroke aphasia at least 4 months prior to randomization and were aged 18 years or older. Participants were excluded if they required treatment in a language other than English, were already using computer therapy to address their word-finding impairment, or had another premorbid speech and language disorder caused by a neurological deficit other than stroke. Participants were randomized to 1 of 3 groups: computer aphasia therapy plus usual care (CSLT), attention or activity control (puzzle books and regular phone calls) plus usual care, or usual care alone. The CSLT intervention targeted the participant’s word-finding impairment. The intervention consisted of self-managed aphasia therapy using the StepByStep software (version 5; Steps Consulting Ltd), which presents a series of word-finding exercises that can be tailored to the individual’s abilities and allows practice items to be personalized.

**Figure 1:**
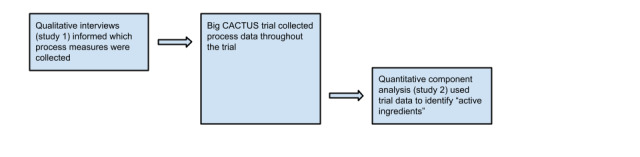
Diagram illustrating the relationship between the qualitative and quantitative studies.

We conducted a multiple methods study within the context of the Big CACTUS randomized controlled trial (see Figure 1). Study 1 comprised qualitative interviews exploring key informants’ understanding of the CSLT intervention, how the components interacted, and how they could be measured. Qualitative findings informed the process measures collected as part of a process evaluation [17] of the CSLT intervention delivered in the Big CACTUS trial. Study 2 comprised quantitative analyses exploring the relationship between intervention process measures and changes in word-finding ability. The methods, analysis, and findings are reported in turn for each study.

### Data and Sample

#### Study 1: Qualitative Interviews

We conducted qualitative interviews with key informants in the early phase of the trial in order to describe the components being delivered in the CSLT approach to word-finding therapy and how the components were thought to interact. We identified the key informants using a nominated expert sampling strategy [18]. The aim was to include a variety of expert perspectives in the sample, including those of (1) the designers of the StepByStep software used in the CSLT approach, (2) therapists who use the CSLT approach regularly in clinical practice, (3) researchers who have evaluated the CSLT approach, (4) patients and caregivers who have helped to design and test the software, as well as (5) representatives of a charity delivering a CSLT service in West Yorkshire. We used a snowball sampling strategy, by asking all participants if they knew anyone with relevant expertise, to ensure experts not known to the authors were not overlooked.

Participants were contacted via email. Information sheets were provided and written consent was sought. The Consent Support Tool [19] was used to determine the optimum format to present information to people with aphasia. Interviews were conducted in person and over the phone at the convenience of the participant. We used an interview schedule to guide the semistructured interviews with questions about what participants perceived to be the components of the intervention, the theories underpinning the components, how the components interact, and how the components could be measured within the Big CACTUS trial. As well as providing a verbal response to the questions, participants were invited to write down the components, so they could refer back to them and arrange them to demonstrate relationships. Post-it notes were used as a visual aid during the interviews; using paper and pen for face-to-face interviews and “Google Drawings” when the interview was conducted over the phone (shared on Google Drive so the interviewer could see it in real time). The interviews were recorded and transcribed verbatim.

#### Study 2: Quantitative Component Analysis

We used trial data from participants randomized to receive the CSLT intervention for which process and outcome data were available in order to identify which intervention components were directly associated with improved word finding. The dependent outcome variable was a change in word finding of 100 personally relevant, treated words assessed using a picture-naming test at baseline and 6 months. The maximum score was 2 points for each word (2=correct response; 1=correct response following repetition, self-correction, or delay of 5 seconds; and 0=incorrect). The pictures were presented in the assessment section of the StepByStep software.

Process variables relating to the delivery and receipt of the components of the CSLT intervention were informed by the findings from the qualitative interviews described below and measured as part of a process evaluation conducted alongside the trial to measure intervention fidelity. Consequently, the process measures included in the quantitative component analysis will be described in the results of the qualitative interviews.

### Analysis

#### Study 1: Qualitative Analysis

We used a 6-stage process of thematic analysis involving familiarization, iterative development of an initial coding framework, identification of themes, reviewing themes, naming and defining themes, and writing up the findings [20]. Familiarization was achieved through transcribing and reading the interview transcripts. A deductive approach was adopted in the early stage of analysis, meaning the questions from the interview schedule informed the higher-order themes. Subsequently, codes emerged from the data that allowed the exploration of different interpretations of the CSLT approach. Analysis of transcripts and visual data (eg, Post-it note diagrams) were managed in NVivo (version 10; QSR International). The process of reviewing and defining the themes and subthemes resulted in the key components being defined as themes and the development of a diagram depicting the components of the intervention and how they interact.

The initial process of familiarization, coding, and theme identification was conducted by MH, an experienced qualitative researcher. The process of reviewing and defining themes was conducted by all authors to increase the dependability of the findings. A reflective journal was kept during the interview and analysis process to enhance the credibility of the research.

#### Study 2: Quantitative Analysis

First, we examined whether individual process variables relating to the components of the intervention were associated with a change in word-finding ability using correlations, 2-tailed *t* tests, and ANOVAs for continuous, binary categorical, and categorical variables, respectively. These bivariate analyses determined the variables for inclusion in a multivariate model using a *P* value cut-off of *P*<.05. Second, we conducted multivariate linear regression analysis examining the association between those process measures identified in the bivariate analyses and change in word-finding ability. The model was adjusted for age and sex. We conducted collinearity checks, and 1 variable was removed where significant multicollinearity was identified. We regarded *P*<.05 as statistically significant. All statistical analysis was conducted using IBM SPSS Statistics for Windows (version 25; IBM Corp).

### Ethical Considerations

Informed consent was obtained from all qualitative study participants, and this element of the study was approved by the Research Ethics Committee in the School of Health and Related Research at the University of Sheffield (002436). For the quantitative component analysis, the following research ethics committees approvals included the secondary analysis of anonymized data without additional consent: Leeds West National Health Service Research Ethics Committee (13/YH/0377) and the Scottish A Research Ethics Committee (14/SS/0023). All data are anonymized. However, in the qualitative study, it may be possible for participants to be identified due to their roles in relation to the StepByStep software; this was highlighted to participants prior to their participation. Participants were not compensated for their time.

## Results

### Study 1: Findings From Qualitative Interviews

#### Participants

Of the 8 CSLT approach experts we invited to participate, 7 took part in 5 individual and 1 joint interview. We conducted 3 interviews face-to-face and 3 over the phone. In total, 4 participants were female. The median age of participants was 49 (range 32-56) years.

All participants used the CSLT approach frequently with use varying from daily to biweekly and the median length of CSLT approach use was 7 (range 2.5-15) years. All expert roles identified a priori were included in the sample (StepByStep software designers, therapists using the CSLT approach regularly, researchers involved in evaluating the CSLT approach, patients and caregivers who have used the CSLT approach and helped to test the StepByStep software, and volunteers supporting patients using the CSLT approach through a charity). In total, 5 participants had more than 1 role in relation to the CSLT approach, and participants’ roles are illustrated after each quote using the italicized words above. The designers who have a financial stake in the StepByStep software declared a potential bias. Full details of participants’ demographic information and relationship to the CSLT approach are presented in Table 1.

**Table 1:** Study 1 participant demographic information and relationship to the CSLT^a^ approach.

Self-selected role in relation to the CSLT approach	Age	Sex	Final level of education	Ethnic group	Years working with CSLT approach	Frequency of CSLT approach use	Financial interest in software
Therapist, designer, and researcher	38	Female	Higher degree	White British	5	Weekly	No
Therapist and researcher	32	Female	Degree	White British	6	Weekly	No
People with aphasia	56	Male	Diploma or certificate in higher education	White British	7	Everyday	No
Caregiver and volunteer	54	Female	Diploma or certificate in higher education	White British	7	Biweekly	No
Volunteer	39	Male	Higher degree	White and Asian	2.5	Daily	No
Therapist, designer, and researcher	49	Female	Higher degree	White British	10	Daily	Yes
Designer	49	Male	Higher degree	White British	15	Biweekly	Yes

^a^CSLT: Computerized Speech and Language Therapy.

#### Findings

**Figure 2:**
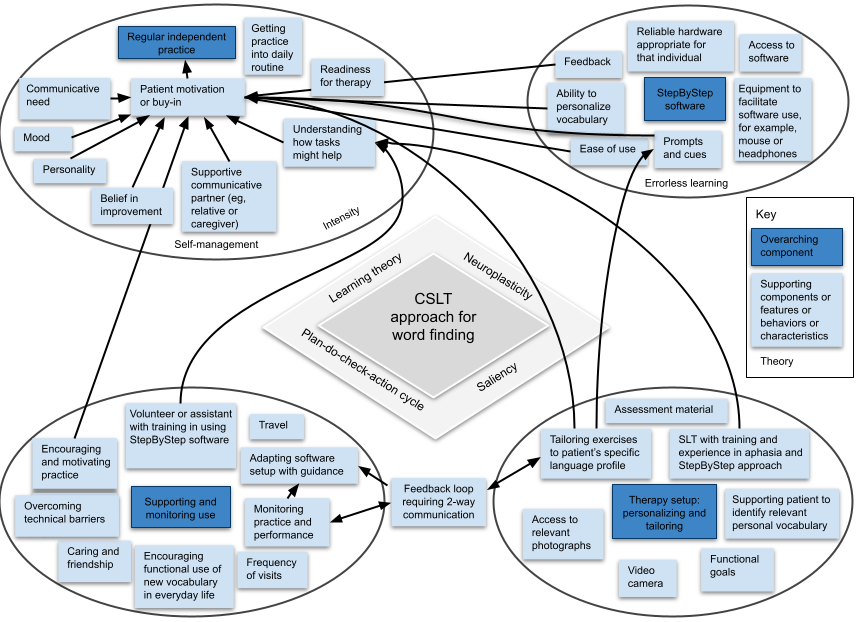
Diagram illustrating the CSLT approach as described by CSLT approach experts. CSLT: Computerized Speech and Language Therapy; SLT: speech and language therapist.

We identified 4 themes describing the overarching components of the CSLT approach: (1) the StepByStep software, (2) therapy setup: personalizing and tailoring, (3) regular independent practice, and (4) supporting and monitoring use. The overarching components are shown in Figure 2 along with the supporting components, features, behaviors, characteristics, and theories that were perceived by participants to influence the delivery of this complex intervention; these will be described in turn below. A final theme related to measuring the components and processes of the CSLT approach.

#### The StepByStep Software

A volunteer described in lay terms their understanding of the StepByStep software:

StepByStep…is a conversion of genuine speech and language therapy exercises that were paper based and have been made into something that can be used on a computer.

Volunteer

Dialogue concentrated on the availability of the software and features of the StepByStep software that motivated use, including perceived ease of use, the prompts and cues available, the feedback the software provides on practice time and performance, and the capacity to personalize vocabulary through selecting items of personal relevance. The importance of having appropriate and reliable hardware and additional equipment, such as a mouse or a microphone, to facilitate software use despite other disabilities, was recognized.

The key theory underpinning the StepByStep software itself is that of errorless learning [21]. The stepped approach starts with tasks that can be more easily achieved before moving on to more difficult exercises, however, this can be enhanced through SLT involvement in tailoring the software.

Although that errorless learning and that stepped approach is still built into the software, so to an extent it would do it on its own, but perhaps we can do it even more sensitively if there’s a speech and language therapist involved.

Therapist, researcher, and designer

#### Therapy Setup: Tailoring and Personalizing

The type and level of difficulty of the exercises on the StepByStep software can be tailored to the patient’s needs. Establishing the most appropriate level of difficulty was perceived to require “formal and informal assessment” of the individual’s language impairment. Tailoring the exercises was perceived by most participants to increase the effectiveness of the therapy because it would “motivate practice and stimulate learning.” Value was placed on the knowledge and skill of the person tailoring the therapy.

You would need a speech and language therapist with some experience in diagnosing and identifying somebody’s level of impairment and then experience and knowledge in how to tailor the program.

Therapist and researcher

An interaction was noted between tailoring the exercises and the monitoring and supporting use component because it is only through monitoring use that suboptimal tailoring can be identified and adjusted. Where tailoring and monitoring are performed by different people this creates an additional requirement for a “feedback loop” between the therapist and the volunteer or assistant.

While the software provides the facility to personalize the vocabulary, the addition of vocabulary relevant to the individual patient was described as part of the therapy setup process. Patient and caregiver participants felt that familiarity enabled the patient to recognize items more easily making the relearning process easier.

Because you couldn’t tell what the picture was, whereas because it’s a photograph you know exactly what it is and if it’s your own kettle then it takes away some of the confusion because it’s something you’re familiar with.

Caregiver and volunteer

#### Regular Independent Practice

As a self-managed intervention, participants perceived regular, repetitive, independent practice to be an essential component of the intervention to enable the desired outcome to be achieved. All of the participants recognized the importance of the patient being motivated or “buying-in” to the CSLT approach to ensure that regular independent practice occurs. Some participants discussed motivation in relation to the individuals’ personality or linked to other internal factors, such as mood or a need for greater communication.

This therapy is hard therapy and so if their communicative need isn’t there then I find that they’re not going to be as motivated and they’re not going to do it as intensively as is required really.

Therapist, researcher, and designer

Whereas others focused on how external factors related to the intervention can influence motivation, for example, the therapist and supporters were perceived to play a key role in ensuring the therapy was set up appropriately, explaining how the intervention works and the process of recovery as well as providing positive or negative reinforcement. The importance of practice being carried out regularly was discussed in relation to the theory of neuroplasticity [22].

I consider the brain to be a muscle and if you were training any other muscle then you’d have to do repeated exercise on that muscle and you’d have to increase, if we talk about weights for example if you were doing a bicep exercise you’d over time have to increase the difficulty by increasing the weight or the repetition to actually have an impact.

Volunteer

#### Supporting and Monitoring Use

Supporting and monitoring the use of the software was the component with the widest variety of interpretations with some debate around who should be providing the support (assistant, volunteer, or therapist). However, irrespective of who was providing the support there was agreement around the activities required to support use and it was acknowledged that this depended on the needs of the individual patient. Activities included enabling the patient to use the software by helping to overcome technical barriers, building a supportive motivational professional relationship or friendship, monitoring practice and performance, adapting the software when required, and enabling opportunities to practice words targeted by the program in conversation in order to aid generalization.

It’s really just giving encouragement and trying to stop there being problems so that the person who’s using it hasn’t got the problems to sort out really, being one step ahead.

Volunteer and caregiver

Someone going along regularly and being someone that cares, I think that in itself, just someone that cares and forming a friendship.

Volunteer

Views regarding the frequency with which support was required varied among participants, but there was agreement about the importance of providing support when needed by the patient and this was perceived to require more frequent visits initially with the frequency diminishing over time for most patients.

It is individualized, as a rule of thumb quite regularly for the first 4-6 weeks and then spread out to once a month.

Volunteer

#### Measuring the Components and Processes of the CSLT Approach

Most participants referred back to their CSLT approach diagram when they described how they would measure the process of delivering the CSLT approach in the Big CACTUS trial. All participants recommended measuring the delivery of some aspect of each of the 4 overarching components. The 10 most frequently described aspects of the CSLT intervention that were perceived to need measuring were selected to be applied to the delivery of the CSLT approach within the Big CACTUS trial, see Table 2.

**Table 2:** Aspects of the intervention participants most frequently described measuring in order to understand the process of delivering and receiving the CSLT^a^ approach in the Big CACTUS trial.

Overarching component	Ten aspects of the CLST approach to be measured in the Big CACTUS trial
StepByStep software	How long was the software available to people with aphasia? How easy is it to use the software?
Therapy setup (tailoring and personalizing)	What sequence of steps are selected and why (eg, justification for tailoring)? How skilled is the person assessing people with aphasia and setting up the software? How are the steps adjusted or adapted in response to the performance of people with aphasia? How much have the practice words been personalized?
Regular independent practice	How much do people practice? How motivated are the people with aphasia to practice? What therapy exercises do people practice?
Supporting and monitoring use	How good is the relationship between the supporter (volunteer or assistant) and the people with aphasia?

^a^CSLT: Computerized Speech and Language Therapy.

The findings from the qualitative element of the study informed the process measures that were collected as part of the fidelity assessment of the Big CACTUS trial and therefore available for inclusion in the quantitative component analysis. The process measures were operationalized as follows:

First, measures related to the StepByStep software included duration of software availability recorded by the therapist (days) and a patient-reported measure of ease of use of the software (how easy is it to use the StepByStep computer therapy? Scored from 1 to 10; 1=very easy).

Second, measures of therapy setup (tailoring and personalizing) included duration of therapist time setting up and supporting the patient (minutes); therapist knowledge of the CSLT approach assessed by a quiz completed 5 months after randomization of their first participant (maximum score=15); completeness of therapists rationale for tailoring the therapy exercises as documented on a therapy planning form (0=partially complete; 1=rationale provided for every exercise); patient-reported measure of personalization of target words (are the words on the StepByStep computer therapy words you want to say? Scored on a 5-point scale from “All” to “None”; 1=all).

Third, regular independent practice measures included duration of therapy practice recorded electronically by the software within 6 months of randomization (minutes); the amount of time spent on the different types of therapy exercises available within the software (picture recognition, confrontation naming, using writing to cue naming, naming from a grid, and naming words in functional sentences) recorded electronically; patient-reported measure of motivation to carry out independent practice (how motivated are you to practice your StepByStep computer therapy exercises? Scored from 1 to 10; 1=very motivated). The “picture recognition” exercise is designed for familiarization with the target words or images. The “confrontation naming” exercise presents the patient with an image of the target word with cues. The “using writing to cue naming” exercise shows a word to spell or an anagram to unscramble in order to prompt retrieval of the word using the voice recognition function. The “naming from a grid” exercise requires the patient to name the items without cues using the speech recognition function and then name the same items again from memory. The “using words in functional sentences” exercise asks a question and requires the patient to answer the question using the target word in a sentence.

Fourth, measures of supporting and monitoring use included duration of support and monitoring reported by the therapy assistant or volunteer (minutes); and the quality of the relationship between the therapy assistant or volunteer and the patient. The quality of the relationship was measured using the Working Alliance Inventory–Short Revised Therapist version (WAI-SRT), which was completed by the assistant or volunteer 4 months into the intervention period [23]. A composite score was used including questions relating to the therapeutic bond, as well as task and goal agreement.

### Study 2: Results of Quantitative Component Analysis

#### Participants

Data from all participants randomized to the intervention arm of the Big CACTUS trial with baseline and 6-month word-finding assessments were included in the analysis (n=83). Due to death, investigator decision, and withdrawal of consent, 14 intervention arm participants did not complete the 6-month outcome measure. Of the 83 participants eligible for inclusion in the quantitative analysis, the mean age was 64.35 years and 57% (n=47) were male. Participant’s mean change in word finding over the 6-month intervention period was 32.84 (SD 30.51).

#### Bivariate Analysis

Process variables relevant to each of the 4 key components highlighted in the qualitative interviews will be described in turn along with their relationship to change in word-finding ability.

The StepByStep software was available to patients for 75.4% (median 138/183 days) of the maximum possible time, and its availability demonstrated a positive, statistically significant correlation with the change in word-finding ability (*r*=0.353; n=83; *P*=.001). The software was perceived to be moderately easy to use (median score 5 out of 10; 1=very easy); however, the number of responses to this question was low, and it was not found to be correlated with change in word-finding ability (*r*=0.245; n=30; *P*=.19).

The median duration of therapist time spent on therapy setup and support was 7 hours 35 minutes; this was not found to be associated with a change in word-finding ability (*r*=0.175; n=83; *P*=.11). The median score on the quiz of the treating therapists’ knowledge of the intervention was 10 out of 15; this was not correlated with a change in word-finding ability (*r*=0.061; n=83; *P*=.581). The rationale for tailoring documented on the therapy planning form was complete for 65% (54/83) of participants compared to having only been partially completed for the remaining participants; the provision of a thorough rationale for tailoring was found to be associated with a change in word-finding ability (*t_80_*=–2.139; n=82; *P*=.04). Along with tailoring, the other aspect of the therapy setup was personalization. Participants perceived that most of the words (median 4 out of 5; 1=none) were of personal relevance; however, the number of responses to the question was low and no association was found (*r*=0.138; n=32; *P*=.67).

The median amount of independent practice carried out by the patients was 25 hours 57 minutes. A weak, positive statistically significant correlation was found between the total amount of practice and change in word-finding ability (*r*=0.271; n=83; *P*=.01). Furthermore, the amount of time spent on 2 of the individual therapy exercises was found to be associated with a change in word-finding ability, with a median of 5 hours 32 minutes spent on confrontation naming exercises (*r*=0.241; n=79; *P*=.03) and 1 hour 38 minutes spent on naming words in functional sentences (*r*=0.313; n=79; *P*=.005). In contrast, no statistically significant association was found between time spent on the remaining 3 exercises and change in word-finding ability, with a median of 1 hour 31 minutes spent on picture recognition or matching exercises (*r*=–0.029; n=79; *P*=.80), 9 hours 7 minutes spent on using writing to cue naming exercises (*r*=0.103; n=79; *P*=.37), and 1 hour 3 minutes spent on naming from a grid exercises (*r*=0.215; n=79; *P*=.06). Participants rated their motivation to carry out regular independent practice as a median of 4 out of 10 (1=very motivated); however, the number of responses was low, and no statistically significant association was found between patients’ level of motivation and change in word-finding ability (*r*=–0.029; n=30; *P*=.88).

The median amount of support and monitoring provided by volunteers and therapy assistants was 4 hours 15 minutes; a weak, positive, and statistically significant correlation was found between the duration of support and monitoring and patients’ change in word-finding ability (*r*=0.286; n=75; *P*=.01). The quality of the relationship between the volunteer or therapy assistant scored using the WAI-SRT scored a median of 4 out of 5; however, the number of responses was low, and no association was found between the WAI-SRT score and change in word-finding ability (*r*=0.183; n=19; *P*=.45).

#### Multivariate Analysis

We entered the process variables found to be associated with a change in word-finding ability into a multivariate linear regression model. Collinearity checks demonstrated that total practice time was highly correlated with time spent practicing 2 of the individual exercises (confrontation naming and naming words in functional sentences), so it was subsequently excluded from the model as the latter 2 variables conveyed more detail. After adjusting for age and sex, we found that providing a thorough rationale for tailoring the therapy exercises (*P*=.04) and time spent practicing naming words in functional sentences exercises (*P*=.046) were statistically significantly associated with change in word-finding ability (see Table 3). The adjusted *R*^2^ was 0.216, indicating that 21.6% of the variance in change in word-finding ability could be accounted for by this multivariate linear regression model.

**Table 3:** Multivariate linear regression on change in word-finding ability^a^.

Variable	Coefficient	*P* value
Duration of software availability (days)	0.141	.25
Completeness of therapist rationale for tailoring the therapy exercises (0=partial; 1=complete)	0.221	.046
Time spent practicing confrontation naming exercises (hours)	0.131	.24
Time spent practicing naming words in functional sentence exercises (hours)	0.236	.04
Duration of support and monitoring reported by the therapy assistant or volunteer (minutes)	0.173	.15
Age (1-year increments)	–0.146	.18
Sex (0=male, 1=female)	–0.1	.35

^a^The value of adjusted *R*^2^ for this multivariate linear regression model is 0.216.

## Discussion

### Key Findings

Through qualitative interviews, 4 overarching components of the CSLT approach for word finding used in the Big CACTUS study were identified: (1) the StepByStep software, (2) therapy setup: tailoring and personalization, (3) regular independent practice, and (4) supporting and monitoring use. In addition to identifying the overarching components of the intervention and providing a detailed description of the underpinning theories, mechanisms, and processes, participants also identified key processes of the CSLT approach that should be measured in the Big CACTUS trial. Through analysis of the association between process and outcome data, 2 “active ingredients” of the CSLT intervention were identified: therapists tailoring the therapy exercises based on the impairment of people with aphasia and people with aphasia practicing naming words in functional sentences.

This study provides a comprehensive description of the CSLT approach from a variety of different perspectives. The complexity of the approach can be seen in the diagram depicting its overarching components and the objects, processes, behaviors, and theories that underpin the intervention. Hawe [24] proposed that complexity can be a property both of an intervention and the context in which it is operationalized and that we can only truly understand that complexity by recognizing that knowledge generation comes from clinicians and implementers as much as it comes from intervention researchers. Thus, the importance of engaging with a variety of stakeholders to truly understand the complexity of a self-managed computer-based intervention, such as the CSLT approach, is highlighted.

The completion of this research also informed the Big CACTUS trial team’s understanding of the CSLT approach and the completion of the TIDieR checklist for the Big CACTUS trial [5,7]. Improved intervention reporting of the CSLT approach has the potential to enable SLTs to deliver this computerized therapy as intended and thereby enable people with aphasia to achieve improved word finding as found in the Big CACTUS trial.

Secondary analysis of data from the Big CACTUS trial identified 2 “active ingredients” of the CSLT approach: therapists tailoring the therapy exercises and people with aphasia practicing using words in functional sentences. As therapists implement the CSLT approach with inevitable adaptations to the local context [12], it may be most beneficial to people with aphasia if they are mindful of the importance of these aspects of the intervention and seek to retain them when adapting the approach locally.

Tailoring the therapy exercises for the individual patient in the CSLT approach involved selecting prompts and cues (eg, semantic or phonological cues) most likely to help the patient based on their impairment. The tailoring of the type and difficulty of therapy exercises is supported by evidence of the effectiveness of model-oriented aphasia therapy, which tailors exercises based on the patient’s symptoms [25]. SLTs have identified that tailoring computer software to the needs of individuals can be time-consuming [26]. It is therefore possible that therapists could be tempted not to tailor in order to offer people with aphasia computerized SLT exercises more time efficiently. However, it would appear to be worth the initial outlay of time as participants’ word finding improved significantly more if a more thorough rationale for tailoring was provided.

The amount of time spent using the computer software to practice naming words in functional sentences was found to be significantly associated with improved word finding. Evidence from the neuroscience literature suggests that it is beneficial to practice the language in relevant action contexts (referred to as the behavioral relevance principle [27]), this could provide a possible explanation for why “naming words in functional sentences” (which sometimes included an action, eg, “Where do you go to do your shopping? We go to the supermarket”) was the only exercise associated with improved word finding. A wide variety of mobile apps for word finding are available on the market, and this finding suggests that being able to practice in sentences may be a key feature to look for in order to maximize improvement of word retrieval [28].

Complex interventions are not static and they do not operate within a vacuum [29]. The StepByStep software itself is constantly being refined and updated (Steps Consulting Ltd website). The findings of this study have resulted in updates to the StepByStep software that encourage people with aphasia to move through the exercises more quickly to increase the amount of time spent on the final exercise, naming words in functional sentences. In addition, a series of apps are in development by Steps Consulting Ltd, including a telehealth app that will allow SLTs to tailor therapy exercises remotely.

Those who have called for more research to investigate the mechanisms between intervention delivery and outcome suggest that one of the benefits of identifying causal components or active ingredients of the intervention is to enable the intervention to be adapted to the local context while remaining effective [11,13]. Therefore, if the components whose delivery is associated with improved outcomes are not delivered, one would not expect the desired outcome to be achieved. As such, the clinical recommendations to therapists implementing the StepByStep approach intervention in clinical practice would be to (1) have a thorough rationale for how the therapy is tailored to the individual people with aphasia and (2) encourage people with aphasia to focus on practicing therapy exercises requiring the production of words in functional sentences.

### Limitations

An interview approach was selected for practical reasons due to participants’ geographical diversity and limited availability and in order to facilitate the involvement of people with aphasia. Focus groups would have provided an opportunity to illuminate agreement and inconsistencies in participants’ understanding of the key components of the CSLT approach [30]. It is possible that the nominated expert sampling strategy did not reach saturation for the qualitative interviews and that there were other StepByStep experts not known to the author or identified by the snowball sampling technique.

Process measures included the use of unvalidated tools to measure tailoring, therapist knowledge, motivation, ease of use, and personalization—a limitation widely acknowledged in fidelity research due, in part, to the need for measures to be tailored to specific interventions [31,32]. Furthermore, it was not possible to measure all processes directly, and consequently, proxy measures were used. For example, due to resource limitations, it was not possible to determine for each person with aphasia the extent to which the therapy program was tailored to the individual’s impairment, and consequently, a proxy measure of providing a thorough rationale for tailoring a therapy planning form was used.

The use of bivariate analyses to determine factors for inclusion in a multivariate model increases the chance of a type I error, due to the model being overfitted. However, the method was used because it offers increased transparency compared to using an automated regression function, such as Stepwise regression [33]. The multivariate model only accounted for 21.6% of the variation in word-finding ability, thus demonstrating that other factors are at play. Possible factors might include individual differences, such as lesion size [34]; wider contextual factors, such as social support; or other components of the intervention not measured within this study. Future research could develop a more comprehensive model to account for improvement in word finding incorporating individual differences, such as those described above, as well as aspects of intervention delivery.

### Conclusions

The qualitative exploration of the CSLT approach provided a detailed description of the components, theories, and mechanisms underpinning the intervention and facilitated the identification of appropriate process measures to be collected in the Big CACTUS trial. The quantitative component analysis furthered our understanding of which components of the intervention are associated with clinical improvement. In order to optimize the benefits of using the CSLT approach for word finding, therapists are advised to pay particular attention to the active ingredients of the intervention: tailoring the therapy exercises based on the individual’s specific language difficulties and encouraging people with aphasia to practice using words in functional sentences.

## Abbreviations

Big CACTUS: Clinical and Cost Effectiveness of Computer Treatment for Aphasia Post Stroke
CSLT: Computerized Speech and Language Therapy
SLT: speech and language therapist
TIDieR: Template for Intervention Description and Replication
WAI-SRT: Working Alliance Inventory–Short Revised Therapist

## Data Availability

The data sets generated and analyzed during this study are available from the corresponding author on request.
